# IRE1 Phosphatase PP2Ce Regulates Adaptive ER Stress Response in the Postpartum Mammary Gland

**DOI:** 10.1371/journal.pone.0111606

**Published:** 2014-11-04

**Authors:** Shuxun Ren, Gang Lu, Asuka Ota, Z. Hong Zhou, Thomas M. Vondriska, Timothy F. Lane, Yibin Wang

**Affiliations:** 1 Department of Anesthesiology, Division of Molecular Medicine, David Geffen School of Medicine, University of California Los Angeles, Los Angeles, CA, United States of America; 2 Departments of Physiology and Medicine, Cardiovascular Research Laboratories, David Geffen School of Medicine, University of California Los Angeles, Los Angeles, CA, United States of America; 3 Departments of Obstetrics and Gynecology and Biological Chemistry, David Geffen School of Medicine, University of California Los Angeles, Los Angeles, CA, United States of America; 4 Departments of Microbiology, Immunology and Molecular Genetics, David Geffen School of Medicine, University of California Los Angeles, Los Angeles, CA, United States of America; University of Texas Health Science Center, United States of America

## Abstract

We recently reported that the *PPM1l* gene encodes an endoplasmic reticulum (ER) membrane targeted protein phosphatase (named PP2Ce) with highly specific activity towards Inositol-requiring protein-1 (IRE1) and regulates the functional outcome of ER stress. In the present report, we found that the PP2Ce protein is highly expressed in lactating epithelium of the mammary gland. Loss of PP2Ce *in vivo* impairs physiological unfolded protein response (UPR) and induces stress kinase activation, resulting in loss of milk production and induction of epithelial apoptosis in the lactating mammary gland. This study provides the first *in vivo* evidence that PP2Ce is an essential regulator of normal lactation, possibly involving IRE1 signaling and ER stress regulation in mammary epithelium.

## Introduction

During lactation, mammary gland produces large quantity of protein and lipid in a timely and quantitatively regulated manner. Mammary gland epithelial cells undergo proliferative and hypertrophic response rapidly at the onset of lactation with dramatically induced protein/lipid synthesis and secretion capacities. The endoplasmic reticulum (ER) is the key organelle for protein and lipid synthesis, secretion and post-translational modification [1]–[3]. Therefore, it is expected that the ER load would be significantly increased in the mammary epithelium during lactation. Overloaded ER would lead to an increase in unfolded or misfolded ER proteins which are able to trigger specific downstream signaling collectively described as the unfolded protein response (UPR). The physiological aspect of UPR is accomplished by compensatory induction of ER chaperones (thus increasing ER capacity) and inhibition of protein translation (thus reducing ER load) in order to restore ER homeostasis [4], [5]. The underlying molecular mechanisms of UPR signaling involve several trans-membrane sensing molecules, including inositol-requiring protein-1 (IRE1), PRKR-like Endoplasmic Reticulum kinase (PERK) and Activating Transcription Factor 6 (ATF-6) [4], [6], [7]. However, abnormal UPR as in the case of persistent ER overload, oxidative injury, and other pathological conditions can trigger cellular dysfunction and apoptosis (Refs). Indeed, abnormal UPR has already been implicated in CNS diseases, diabetes, obesity, inflammation and heart diseases [8]–[13]. It is well recognized that lactation involves abrupt changes in ER load in mammary gland epithelium, and ER stress related genes have been shown to be dynamically expressed during the lactation cycle [14]. However, the importance of ER stress signaling in normal mammary gland physiology has not been demonstrated.

In UPR signaling, IRE1 activity has the unique ability to stimulate multiple pathways, one with adaptive, the other with detrimental, effects in cells. Oligomerization of IRE1 activates RNase activity and downstream splicing of X-box binding protein 1(XBP1) mRNA as well as degradation of 28S rRNA. XBP1 acts as a transcription factor to induce chaperone protein expression; loss of 28S rRNA due to IRE1-dependent RNAse activity leads to inhibition of protein synthesis [15]–[19] [20]. On the other hand, IRE1 activation can also cause cell death via TRAF2-ASK1-JNK pathway [21]–[24]. A key step in IRE1 activation is the oligomerization and subsequent trans-autophosphorylation. Recently, we discovered that an ER localized protein phosphatase, PP2Ce, dephosphorylates IRE1 with high selectively, resulting in a potent IRE1 inhibitory effect [25]. The PP2Ce coding gene, *ppm1l*, is significantly associated with obesity and metabolic disorder based on genome wide association studies [26] [25], providing genetic evidence for a link between IRE1 regulation and metabolic diseases. However, specific contribution of PP2Ce in mammary gland function and ER stress regulation has not been investigated.

In the present study, we found that PP2Ce was significantly expressed in mammary gland epithelium and dynamically regulated at the onset of lactation, correlating with similar expression profile of IRE1α. Genetic inactivation of PP2Ce in female mice caused lactation defects associated with abnormal UPR and enhanced stress signaling. In addition, abnormal ER ultra-structure and a significant induction of apoptosis was detected in the lactating mammary epithelium of PP2Ce deficient mice comparing the wildtype controls. Therefore, this study shows for the first time a critical function for PP2Ce in UPR and lactation in mammary gland.

## Materials and Methods

### Animals

The *ppm1k* knockout and wildtype mice were established in C57BL6 background as described [25]. Specifically, a IRES-neo-lacZ cassette was inserted at mouse *ppm1l* exon 4 region, replacing 183 bp (of the coding sequence while directing the lacZ expression by the *ppm1l* locus. The knockout allele was identified by genomic DNA PCR for genotyping using a pair of oligoes, A1 for mouse genomic sequence and A2 for Neo cassette sequence (see below for sequences). A1: GCCTCTGTAAAAGGACTGCAGGACG. A2: GGGTGGGATTAGATAAATGCCTGCTCT. Homozygous KO mice were generated by breeding two heterozygous KO mice and maintained by breeding homozygous KO males with heterozygous KO females. All animals were maintained and bred at certified animal facility of University of California, at Los Angeles. All animal work were conducted following protocols approved by UCLA Office of Animal Research Oversight.

### Immunoblotting

Polyclonal antibody against β-Actin, p-JNK, JNK, p-p38, p38, caspase-12 and activated caspase-12 were purchased from Cell Signaling and used following manufacturer's recommendations. Tissues were harvested and homogenized in Buffer (50 mM HEPES [pH 7.4], 150 mM NaCl, 1% Triton X-100, 1 mM EDTA, 1 mM EGTA, 1 mM glycerophosphate, 2.5 mM sodium pyrophosphate 1 mM Na3VO4, 20 mM NaF, 1 mM phenylmethylsulfonyl fluoride, 1 µg/mL of aprotinin, leupeptin, and pepstatin). Total soluble lysates were subjected to SDS-PAGE on 6%Tris-Glycine, or 4–12% Bis-Tris or 12% Bis-Tris gels and analyzed with indicated primary antibodies. Protein signals were detected using enhanced chemiluminescence (ECL) western blotting detection regents (Pierce).

### Realtime RT-PCR analysis

Total RNA was extracted from tissues using Trizol Reagent (Invitrogen) according to manufacturer's instructions. 5 µg total RNA was reverse transcribed into the first-strand cDNA using Superscript First-strand synthesis Kit (Invitrogen) with oligo-dT primers. Then, cDNA transcripts were quantified by iCycler iQ Real-Time PCR Detection System (Bio-RAD) using iQ SYBR Green Supermix (Bio-RAD). Each reaction was performed in duplicate and values were averaged to calculate the relative expression level. The RT-PCR primers used are listed below: PP2Ce: 5′- TTGTCTCACGATCACAAGCC; 3′- AGATGTCTGGGTCTGGGATG IRE1α: 5′- ACGGTGGACATCTTTTCTGC; 3′- TGGGGATCCATAGCAATCAT EDEM: 5′- TCTGTGGACAAACGTCTTCG; 3′- GGCGCATGTAGATGCTCTTT Bip: 5′- GAGGCTGTAGCCTATGGTGC; 3′- TTTGTTAGGGGTCGTTCACC CHOP: 5′- TATCTCATCCCCAGGAAACG; 3′- GGGCACTGACCACTCTGTTT GAPDH: 5′- TCCTGCACCACCAACTGCTTAG; 3′- GATGACCTTGCCCACAGCCTTG

### Whole-mount carmine and β-galactosidase staining of mouse mammary glands

Female mice were killed around 10 wk, freshly dissected mammary gland tissue were flatly placed on glass slides and fixed in Carnoy's solution (70% ethanol, 20% chloroform, and 10% glacial acetic acid) for 1 hr at room temperature. The fixed glands were washed in 70% ethanol for 15 min and then rinsed in water for 5 min. The mammary glands were stained overnight at 4°C in carmine aluminum solution (1 g carmine, sigma C1022 and 2.5 g aluminum potassium sulfate (Sigma A7167) in 500 ml water). The glands were then dehydrated progressively in 70%–95%–100% ethanol, cleared in xylene for 30 min, and mounted on glass slides with Permount (Fisher Scientific, Suwanee, GA). For β-gal staining, same tissues were 0.2% paraformaldehyde in PBS (0.1 M, PH = 6.9) with 2 mM MgCl_2_ and 5 mM EGTA overnight at 4°C. After washing three times with PBS, the tissue samples were incubated for 2 h at 30°C in X-gal reaction buffer containing 5 mM K-Ferricyanide, 5 mM K-Ferrocyanide, 0.02% NaDecoxycholate, 0.01% NP-40, 2 mM MgCl2 and 1 mg/ml X-gal for 24 to 48 h at 30°C. The tissues were then washed with PBS. The stained tissues were viewed under Nikon SM21500 dissection microscope and images were recorded digitally.

### Histological analysis

Mammary gland samples were dissected from 10 month old virgins or female animals at postpartum day 0 and 2. The tissues were fixed overnight in 10% formalin (SIGMA HT501128-4 L) and processed as following: 40°C in automatic tissue processors through 50, 80, 95, 100% ethanol and xylene, then embedded in paraffin molds at 60°C. Blocks were sectioned in a microtome at 4 µm followed by deparaffinization and rehydration through 3 cycles of xylenes and graded ethyl alcohols to PBS. The sections were blocked 30 min at room temperature in TNB blocking buffer (PerkinElmer, NEL 701) and then incubated with WAP primary antibody (1: 100; Santa Cruz, sc-14832) for overnight. Washed twice with PBS and stained with fluorochrome-coupled anti-goat secondary antibodies (Molecular Probes, A-11055), samples were mounted for microscopic inspection in mounting medium (DAPI, Vector Lab. Inc.). Images were viewed from a Nikon Ccnfocol fluorescent microscope according to manufacturer's instruction.For transmission electron microscopy, the tissues were fixed, sectioned, mounted as described [27] and imaged at UCLA Electron Imaging Center for Nanomachines.

### Statistical Analysis

All statistical analysis was performed using student t-test for comparison between two groups and ANOVA test for multi-group comparisons. p<0.05 was accepted as significant.

## Results

### PP2Ce expression in lactating mammary gland

**Figure 1:**
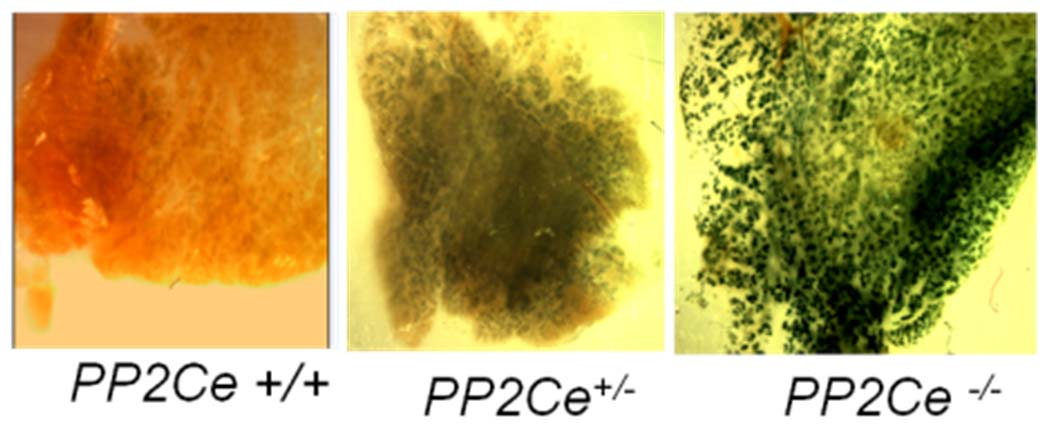
PP2Ce expression in mouse mammary epithelium. Representative images of Lac-Z staining of mammary tissues isolated from 7 to 9 weeks of virgin female wildtype, *PP2Ce*-/+ and *PP2Ce -/-* animals.

**Figure 2:**
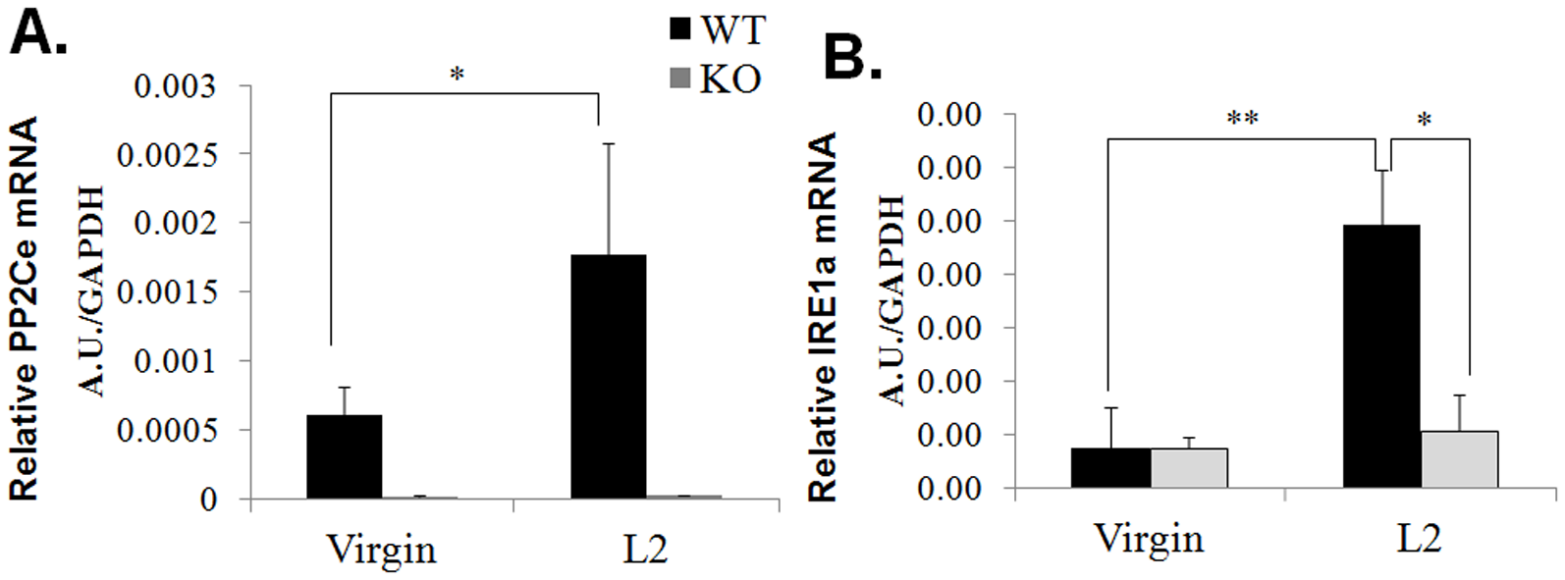
PP2Ce and IRE1α expression in peri-partum mouse mammary gland. **A.** PP2Ce and **B.** IRE1α mRNA in mammary gland as measured by qRT-PCR at postpartum day 0 (L0) and day 2 (L2) in wildtype (WT) and *PP2Ce -/-* (KO) mice.

Using a mouse line with lacZ knocked in at the *ppm1l* locus [25], we first determined the expression of PP2Ce in intact mammary gland using whole-mount X-gal staining. As shown in Figure 1, a high level of X-gal positive staining reflecting the PP2Ce expression pattern was detected in the epithelium of mammary gland. Therefore, *ppm1l*-driven expression of X-gal in the knock-out reveals *ppm1l* expression in the mammary gland, which is confirmed by analysis via qRT-PCR. Using qRT-PCR, we found PP2Ce mRNA levels to be significantly induced in the mammary gland of the postpartum animal as compared with that in virgin females (Figure 2A). In parallel with PP2Ce expression, IRE1α mRNA was also significantly induced in the mammary glands of lactating mice versus virgin females (Figure 2B). Therefore, PP2Ce and IRE1α are both induced at the onset of lactation in postpartum mammary glands.

### PP2Ce is essential for normal lactation

**Figure 3:**
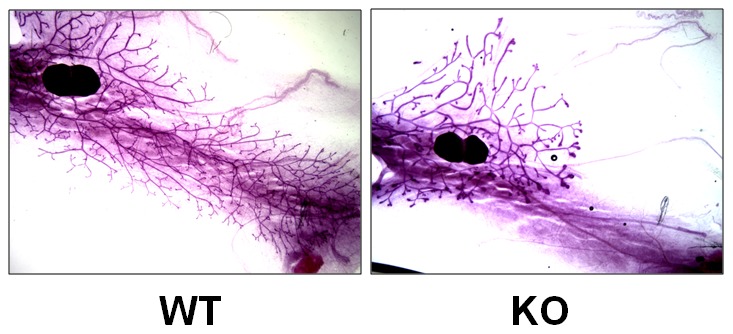
PP2Ce on mammary gland development. Representative whole-mount images of ductal architecture of mammary gland from wild type (WT) and *PP2Ce -/-* (KO) virgin females at around 10 weeks of age illustrated by carmine staining (Methods).

To determine the functional role of PP2Ce in mammary gland function, a genetic knockout mouse line was created as described [25], [26]. Inactivation of PP2Ce expression in mammary gland was validated at mRNA level by qRT-PCR (Figure 2A). In virgin females, histological analysis of mammary gland showed similar gross morphology and branching profile between the wildtype and PP2Ce knockout (KO) mice (Figure 3). The litter sizes produced by the PP2Ce KO females are similar at birth comparing with those produced by the PP2Ce heterozygous females, however, all offspring from the PP2Ce KO females died within two days postnatally regardless of their own genotypes (Table 1). Upon closer inspection, we found that the neonates from the PP2Ce KO mothers died from a lack of milk consumption. Consequently, by switching to foster mothers of wildtype (WT) background, the newborn mice survived at level comparable to that of neonates produced by the heterozygous females (Table 1). This observation suggests that PP2Ce deficiency leads to lactation defects of mammary gland.

**Table 1:** Survival rate of offspring to PP2Ce −/+ and *PP2Ce -/-* females with or without widtype foster mother.

Mother Genotype	PP2Ce +/− (n = 12)	PP2Ce −/− (n = 3)	PP2Ce −/− plus WT foster mother (n = 7)
Survival/Total	54/83	0/21	29/51
Average Litter Size	6.9±1.6	7.0±1.0	7.3±1.6
Average Survival/Litter	4.5±3.2	0	4.1±2.6

### Mammary gland defects in PP2Ce deficient females

**Figure 4:**
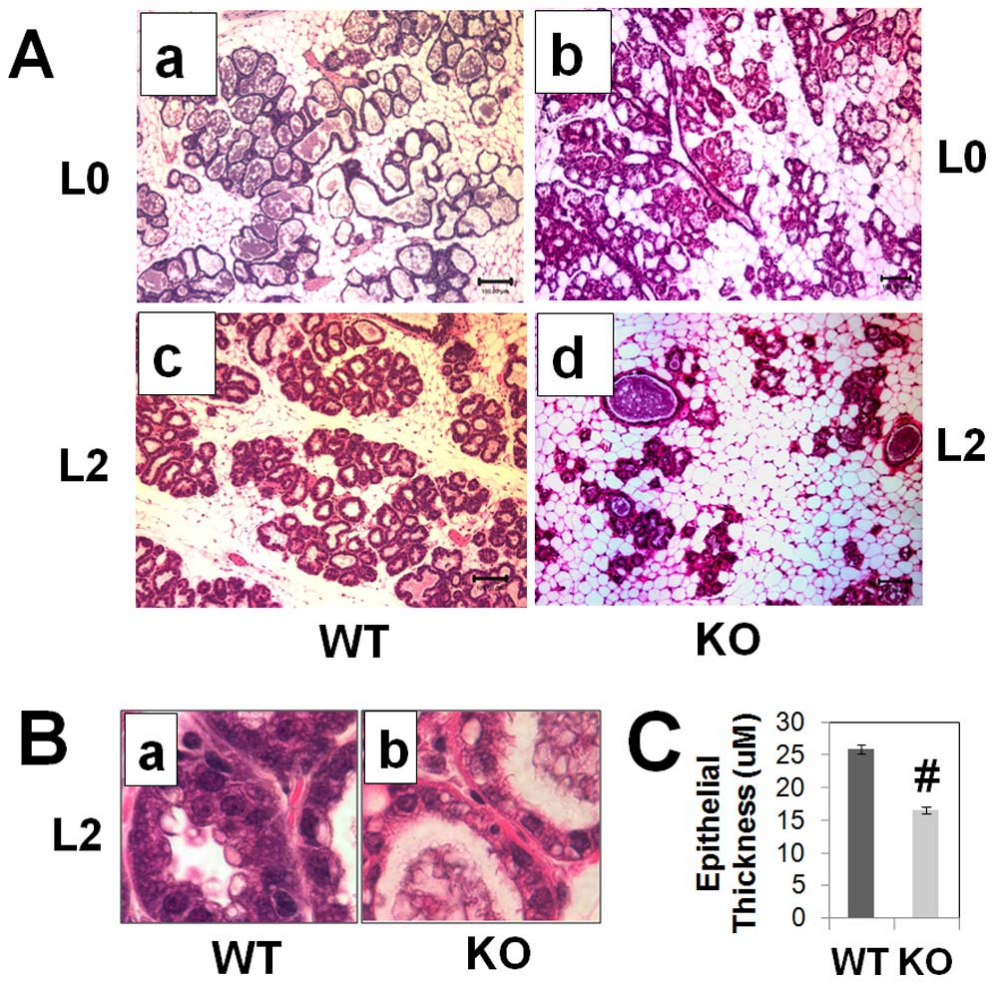
Mammary gland defects in postpartum PP2Ce deficient mice. **A.** Representative H&E images (10X lens) of mammary tissue from wildtype (a and c) and *PP2Ce -/-* (KO) (b and d) at postpartum day 0 (L0) and day 2 (L2), showing reduced alveolar density and regression of epithelium in postpartum KO tissue. **B.** Abnormal mammary epithelium in lactating PP2Ce deficient mice. H&E image of epithelial structure and morphology at higher magnification (60X lens). **C.** Quantitative analysis of epithelium wall thickness in L2 lactating wildtype (WT) and PP2Ce deficient (KO) mammary gland showing average value from 20 sections collected from at least 3 animals in each group. #, p<0.05 KO vs. WT.

**Figure 5:**
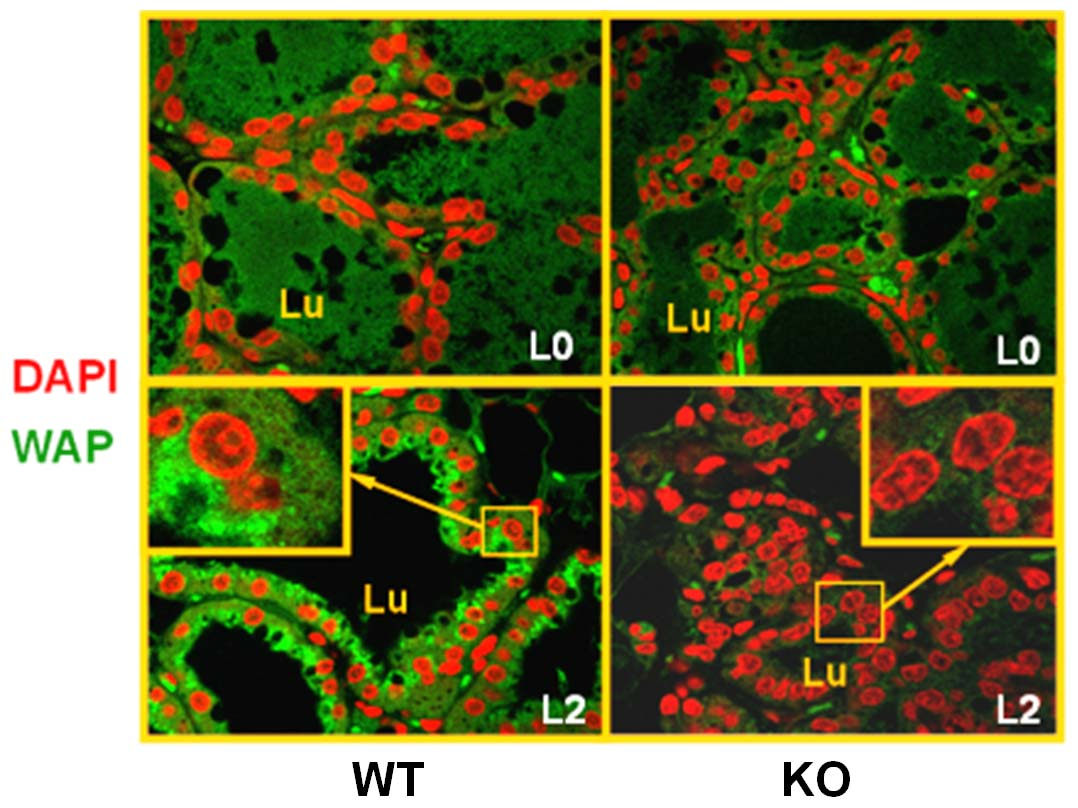
Defects in milk production and secretion in postpartum PP2Ce deficient mice. Representative immunofluorescent images for DAPI (red) and Whey Acid Protein (green) with enlarged region shown in the inserts, in wildtype (WT) and *PP2Ce-/-* (KO) mammary gland at postpartum day 0 (L0) and day 2 (L2). Negative staining for WAP was observed in mammary gland sections from all three L2 PP2Ce mice analyzed.

We examined the mammary gland in the PP2Ce KO and wildtype control (WT) mice at day 0 and day 2 postpartum. As shown in Figure 4A, the PP2Ce KO females have morphologically normal mammary glands as compared with the WT controls at postpartum day 0, consistent with our observation in virgin females. However, at postpartum day 2, the PP2Ce KO mice showed dramatically diminished aveolar presence in the mammary gland. In addition, the thickening of the epithelial layer and secretary vesicles prominent in the WT tissue were significantly reduced and disrupted in the PP2Ce KO tissue (Figure 4B and 4C). By immuno-staining, we observed a major induction of WAP, a major milk component, at the apical layer of mammary epithelium of WT mice at postpartum day 2, whereas the PP2Ce KO tissues were negative for WAP staining (Figure 5). In WT female mice, removing newborns at birth did not change mammary gland morphology or WAP staining at postpartum day 2 as observed in L2 WT females. These data support the conclusion that PP2Ce is required for normal production of milk and maintenance/expansion of the postpartum mammary epithelium.

### PP2Ce KO triggers defective UPR and stress signaling in postpartum mammary gland

**Figure 6:**
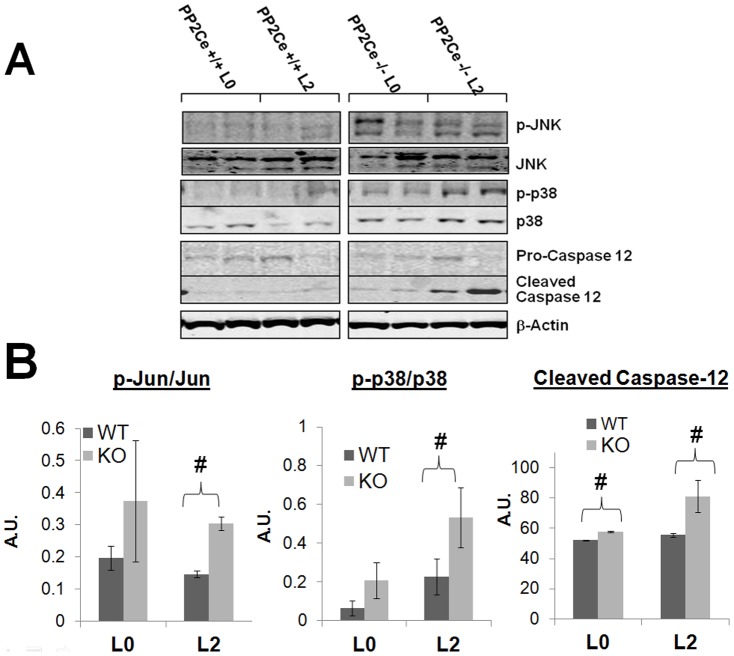
Stress signaling activation in postpartum PP2Ce deficient mammary gland. **A.** Immunoblot of phospho-JNK, phospho-p38 and Caspase 12 activation (cleaved) levels in *PP2Ce +/+* and *PP2Ce -/-* mammary tissue at day 0 (L0) or day 2 post parturition (L2). **B.** Quantification of signaling activation from at least 3 samples from each group. #, p<0.05 WT vs. KO.

**Figure 7:**
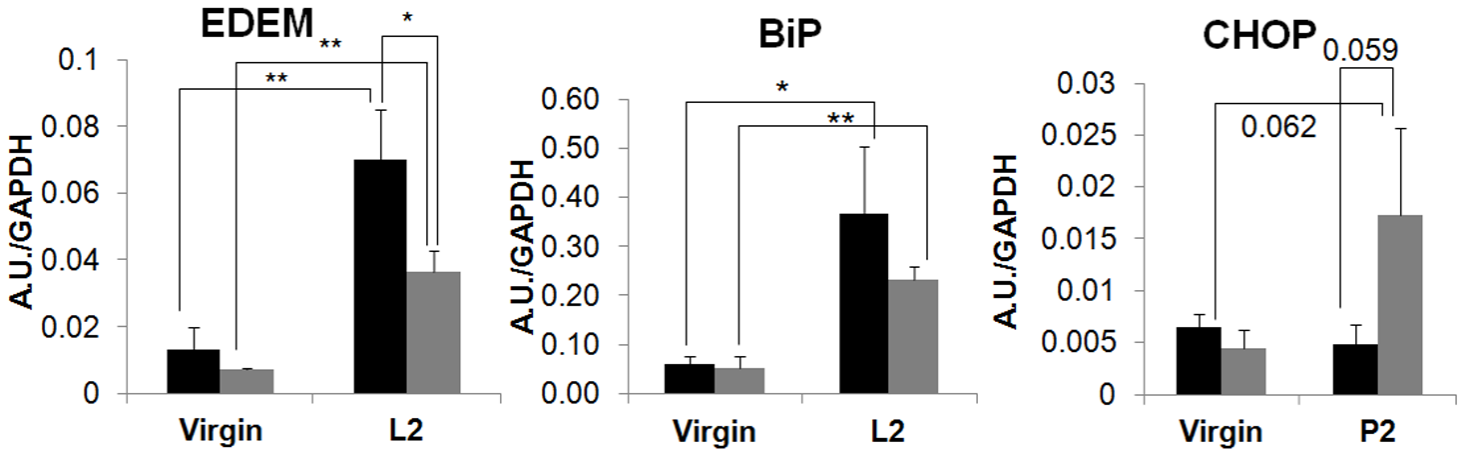
Effect of PP2Ce deficiency on ER stress response. mRNA levels of adaptive ER stress response markers BIP and EMDM, and maladaptive ER stress response marker CHOP in wildtype (WT) and *pp2ce-/-* (KO) mammary tissue at day 0 (L0) and day 2 (L2) postpartum. * p<0.05, ** p<0.01 by ANOVA test.

To determine the underlying mechanisms for the defects observed in PP2Ce KO mammary gland, we examined the effect of PP2Ce deficiency on stress signaling. In agreement with our previous *in vitro* study, where PP2Ce KO MEF cells had elevated stress-kinase signaling upon ER stressor stimulation [25], stress kinases were significantly induced in postpartum PP2Ce KO mammary gland compared to WT controls (Figure 6). Correlated with induced IRE1α expression (Figure 2B), downstream UPR genes including BiP and EDEM are significantly induced in postpartum wildtype mammary gland (Figure 7); induction of these genes, including IRE1α was markedly blunted in the PP2Ce KO mice (Figure 2B and Figure 7). In contrast, there is trend for the induction of pathological ER stress signaling marker CHOP in the mammary gland of postpartum PP2Ce KO mice (Figure 7). All this evidence suggests that PP2Ce is essential for adaptive ER stress response at the onset of lactation and its deficiency can trigger pathological stress signaling in the postpartum mammary gland.

### PP2Ce KO induces apoptotic cell death in postpartum mammary epithelium

**Figure 8:**
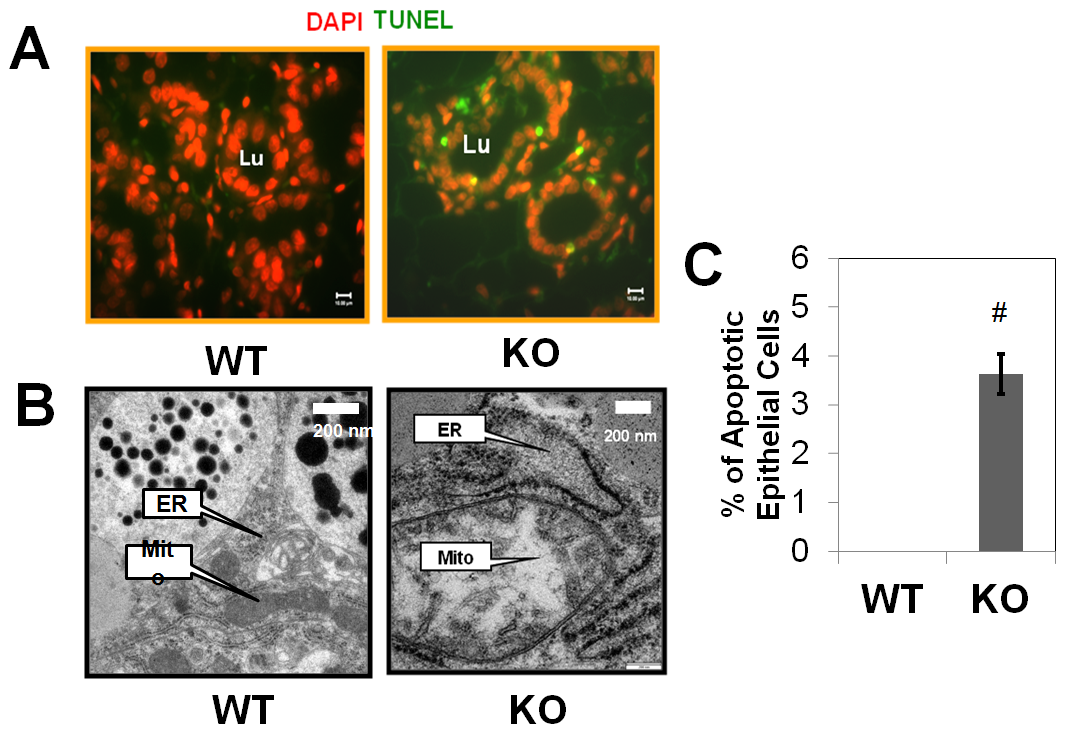
Induced apoptosis in postpartum PP2Ce deficient mammary epithelium. **A.** Immuno-fluorescent images of DAPI (red) and TUNEL (Green) in wildtype (WT) and *PP2Ce-/-* (KO) mammary tissues at postpartum day 2. **B.** Representative electron microscopic images of mammary epithelium in wildtype (WT) and *PP2Ce-/-* (KO) mice at postpartum day 2, showing lipid droplet, nuclei, ribosome decorated rough ER (ER) and mitochondria (mito) as indicated. **C.** Average percentage of TUNEL positive epithelial cells in WT and KO mammary tissue in postpartum day 2. #, p<0.05 WT vs. KO.

It is well established that constitutive activation of IRE1 signaling can trigger apoptosis via caspase-12 activation. In the PP2Ce KO mammary gland, we indeed observed a marked induction of apoptosis based on TUNEL analysis in postpartum tissue (Figure 8A and C), which was associated with caspase-12 activation (Figure 6). Ultra-structure analysis also detected signs of enlarged ER lumen and mitochondrial swelling, supporting the status of pathological ER stress response and potential involvement of apoptosis (Figure 8B). Therefore, PP2Ce is essential for adaptive ER stress signaling, milk production and cellular survival in lactating mammary cells.

## Discussion

ER stress signaling is important in both physiological UPR as well as ER stress-induced programmed cell death. In this study, we found that an IRE1 specific phosphatase, PP2Ce, is essential for normal lactation function in the postpartum mammary gland. A sharp increase in protein and lipid synthesis, as is observed during normal lactation, increases ER load dramatically; therefore, it would be expected that an adaptive ER stress response would be induced in the lactating mammary gland. It is surprising, however, that a detailed characterization and demonstration of the importance of ER stress signaling in the lactating mammary gland has not been reported to date. A recent study in lactating bovine confirmed a coordinated change in ER stress genes during the lactation cycle [14]. In agreement with this observation, we found significant induction of IRE1α and the downstream adaptive genes BiP and EDEM in the postpartum mouse mammary gland. However, these adaptive changes were attenuated in the PP2Ce deficient mice. In contrast, pathological ER stress markers, including CHOP expression, JNK activation and caspase-12 activation were observed in the postpartum PP2Ce deficient mammary gland. To our knowledge, this *in vivo* observation represents the first evidence to implicate the importance of PP2Ce in mammary gland physiology. The correlated changes in ER stress signaling in the PP2Ce deficient mammary gland supports the potential role of PP2Ce as an IRE1 regulator in this process as demonstrated in vitro [25], although other uncharacterized downstream targets of PP2Ce can also contribute to the phenotype observed.

ER stress regulation is important for many physiological functions, including inflammation and metabolism [28]–[30]. Dysfunction of ER stress signaling has been implicated in many human diseases, such as cancer, obesity, diabetes, neurodegenerative diseases and atherosclerosis [31]. In fact, the gene coding for PP2Ce, *ppm1l*, was first identified as candidate disease-causing gene for complex traits associated with metabolic syndrome [26] [25]. Our study adds normal mammary gland function as one more important organ where PP2Ce plays a critical role. This observation fits with the physiological demands on the mammary gland: few if any other tissues endure such a sudden increase (in both magnitude and speed) of protein and lipid synthesis and secretion. As an adaptive process, ER stress signaling functions to meet protein/lipid demand with synthesis capacity, clearly an important requirement for the mammary gland during lactation. It will be interesting to investigate if the PP2Ce signaling plays a role in the gradual increase in milk production as lactation proceeds, as well as the sudden termination of production after weaning. In addition, if ER stress is the primary mechanism affected by PP2Ce, it would be interesting to investigate whether ER stress relieve, such as treating animals with sodium phenylbutyrate would alleviate the defects observed in PP2Ce deficient mice. These questions can also be tested in models where IRE1α-PP2Ce signaling is manipulated *in vivo*. Lactation defects are complex medical conditions that can affect infant development and long term health [32]. With the discovery of PP2Ce as an important molecule and the potential link with ER stress signal regulation in normal milk production in the postpartum mammary gland, our study offers new important molecular insights to mammary gland physiology and disease.

## References

[1] Baumann O, Walz B (2001) Endoplasmic reticulum of animal cells and its organization into structural and functional domains. Int Rev Cytol 205: 149–214. 11336391 10.1016/s0074-7696(01)05004-5

[2] Berridge MJ (2002) The endoplasmic reticulum: a multifunctional signaling organelle. Cell Calcium 32: 235–249. 12543086 10.1016/s0143416002001823

[3] Harding HP, Calfon M, Urano F, Novoa I, Ron D (2002) Transcriptional and translational control in the Mammalian unfolded protein response. Annu Rev Cell Dev Biol 18: 575–599. 12142265 10.1146/annurev.cellbio.18.011402.160624

[4] Ron D, Walter P (2007) Signal integration in the endoplasmic reticulum unfolded protein response. Nat Rev Mol Cell Biol 8: 519–529. 17565364 10.1038/nrm2199

[5] Schroder M, Kaufman RJ (2005) The mammalian unfolded protein response. Annu Rev Biochem 74: 739–789. 15952902 10.1146/annurev.biochem.73.011303.074134

[6] Bertolotti A, Zhang Y, Hendershot LM, Harding HP, Ron D (2000) Dynamic interaction of BiP and ER stress transducers in the unfolded-protein response. Nat Cell Biol 2: 326–332. 10854322 10.1038/35014014

[7] Wu J, Kaufman RJ (2006) From acute ER stress to physiological roles of the Unfolded Protein Response. Cell Death Differ 13: 374–384. 16397578 10.1038/sj.cdd.4401840

[8] Kapoor A, Sanyal AJ (2009) Endoplasmic reticulum stress and the unfolded protein response. Clin Liver Dis 13: 581–590. 19818306 10.1016/j.cld.2009.07.004

[9] Minamino T, Kitakaze M (2010) ER stress in cardiovascular disease. J Mol Cell Cardiol 48: 1105–1110. 19913545 10.1016/j.yjmcc.2009.10.026

[10] Minamino T, Komuro I, Kitakaze M (2010) Endoplasmic reticulum stress as a therapeutic target in cardiovascular disease. Circ Res 107: 1071–1082. 21030724 10.1161/CIRCRESAHA.110.227819

[11] Tabas I (2010) The role of endoplasmic reticulum stress in the progression of atherosclerosis. Circ Res 107: 839–850. 20884885 10.1161/CIRCRESAHA.110.224766 PMC2951143

[12] Thomas SE, Dalton LE, Daly ML, Malzer E, Marciniak SJ (2010) Diabetes as a disease of endoplasmic reticulum stress. Diabetes Metab Res Rev 26: 611–621. 20922715 10.1002/dmrr.1132

[13] Vidal R, Caballero B, Couve A, Hetz C (2011) Converging pathways in the occurrence of endoplasmic reticulum (ER) stress in Huntington's disease. Curr Mol Med 11: 1–12. 21189122 10.2174/156652411794474419

[14] Invernizzi G, Naeem A, Loor JJ (2012) Short communication: Endoplasmic reticulum stress gene network expression in bovine mammary tissue during the lactation cycle. Journal of Dairy Science 95: 2562–2566. 22541483 10.3168/jds.2011-4806

[15] Calfon M, Zeng H, Urano F, Till JH, Hubbard SR, et al (2002) IRE1 couples endoplasmic reticulum load to secretory capacity by processing the XBP-1 mRNA. Nature 415: 92–96. 11780124 10.1038/415092a

[16] Lee AH, Iwakoshi NN, Glimcher LH (2003) XBP-1 regulates a subset of endoplasmic reticulum resident chaperone genes in the unfolded protein response. Mol Cell Biol 23: 7448–7459. 14559994 10.1128/MCB.23.21.7448-7459.2003 PMC207643

[17] Lee K, Tirasophon W, Shen X, Michalak M, Prywes R, et al (2002) IRE1-mediated unconventional mRNA splicing and S2P-mediated ATF6 cleavage merge to regulate XBP1 in signaling the unfolded protein response. Genes Dev 16: 452–466. 11850408 10.1101/gad.964702 PMC155339

[18] Oda Y, Okada T, Yoshida H, Kaufman RJ, Nagata K, et al (2006) Derlin-2 and Derlin-3 are regulated by the mammalian unfolded protein response and are required for ER-associated degradation. J Cell Biol 172: 383–393. 16449189 10.1083/jcb.200507057 PMC2063648

[19] Yoshida H, Matsui T, Yamamoto A, Okada T, Mori K (2001) XBP1 mRNA is induced by ATF6 and spliced by IRE1 in response to ER stress to produce a highly active transcription factor. Cell 107: 881–891. 11779464 10.1016/s0092-8674(01)00611-0

[20] Iwawaki T, Hosoda A, Okuda T, Kamigori Y, Nomura-Furuwatari C, et al (2001) Translational control by the ER transmembrane kinase/ribonuclease IRE1 under ER stress. Nat Cell Biol 3: 158–164. 11175748 10.1038/35055065

[21] Nakagawa T, Zhu H, Morishima N, Li E, Xu J, et al (2000) Caspase-12 mediates endoplasmic-reticulum-specific apoptosis and cytotoxicity by amyloid-beta. Nature 403: 98–103. 10638761 10.1038/47513

[22] Nishitoh H, Matsuzawa A, Tobiume K, Saegusa K, Takeda K, et al (2002) ASK1 is essential for endoplasmic reticulum stress-induced neuronal cell death triggered by expanded polyglutamine repeats. Genes Dev 16: 1345–1355. 12050113 10.1101/gad.992302 PMC186318

[23] Urano F, Wang X, Bertolotti A, Zhang Y, Chung P, et al (2000) Coupling of stress in the ER to activation of JNK protein kinases by transmembrane protein kinase IRE1. Science 287: 664–666. 10650002 10.1126/science.287.5453.664

[24] Yoneda T, Imaizumi K, Oono K, Yui D, Gomi F, et al (2001) Activation of caspase-12, an endoplastic reticulum (ER) resident caspase, through tumor necrosis factor receptor-associated factor 2-dependent mechanism in response to the ER stress. J Biol Chem 276: 13935–13940. 11278723 10.1074/jbc.M010677200

[25] Lu G, Ota A, Ren S, Franklin S, Rau CD, et al. (2013) PPM1l encodes an inositol requiring-protein 1 (IRE1) specific phosphatase that regulates the functional outcome of the ER stress response. Molecular Metabolism. 10.1016/j.molmet.2013.07.005 PMC3854994 24327956

[26] Chen Y, Zhu J, Lum PY, Yang X, Pinto S, et al (2008) Variations in DNA elucidate molecular networks that cause disease. Nature 452: 429–435. 18344982 10.1038/nature06757 PMC2841398

[27] Petrich BG, Gong X, Lerner DL, Wang X, Brown JH, et al (2002) c-Jun N-terminal kinase activation mediates downregulation of connexin43 in cardiomyocytes. Circ Res 91: 640–647. 12364393 10.1161/01.res.0000035854.11082.01

[28] Ron D, Hubbard SR (2008) How IRE1 reacts to ER stress. Cell 132: 24–26. 18191217 10.1016/j.cell.2007.12.017

[29] Schroder M (2008) Endoplasmic reticulum stress responses. Cell Mol Life Sci 65: 862–894. 18038217 10.1007/s00018-007-7383-5 PMC11131897

[30] Zhang K, Kaufman RJ (2008) From endoplasmic-reticulum stress to the inflammatory response. Nature 454: 455–462. 18650916 10.1038/nature07203 PMC2727659

[31] Lin JH, Walter P, Yen TS (2008) Endoplasmic reticulum stress in disease pathogenesis. Annu Rev Pathol 3: 399–425. 18039139 10.1146/annurev.pathmechdis.3.121806.151434 PMC3653419

[32] Bergmann RL, Bergmann KE, von Weizsacker K, Berns M, Henrich W, et al. (2013) Breastfeeding is natural but not always easy: intervention for common medical problems of breastfeeding mothers - a review of the scientific evidence. J Perinat Med: 1–10. 10.1515/jpm-2013-0095 24057589

